# Evaluation of Laser Confocal Raman Spectroscopy as a Non-Invasive Method for Detecting Sperm DNA Contents

**DOI:** 10.3389/fphys.2022.827941

**Published:** 2022-02-08

**Authors:** Mengge Li, Yaxing Ji, Dongmei Wang, Yanliang Zhang, Huan Zhang, Yi Tang, Ge Lin, Liang Hu

**Affiliations:** ^1^National Engineering and Research Center of Human Stem Cells, Changsha, China; ^2^Hunan Guangxiu Hospital, Changsha, China; ^3^NHC Key Laboratory of Human Stem Cell and Reproductive Engineering, Institute of Reproductive and Stem Cell Engineering, School of Basic Medical Science, Central South University, Changsha, China; ^4^Thermo Fisher Scientific, Shanghai, China; ^5^Clinical Research Center for Reproduction and Genetics in Hunan Province, Reproductive and Genetic Hospital of CITIC-XIANGYA, Changsha, China; ^6^Hunan International Scientific and Technological Cooperation Base of Development and Carcinogenesis, Changsha, China

**Keywords:** laser confocal Raman spectroscopy, human sperm, fluorescence *in situ* hybridization, DNA content, preimplantation genetic testing

## Abstract

**Research Question:**

Is Raman spectroscopy an efficient and accurate method to detect sperm chromosome balance state by DNA content differences?

**Design:**

Semen samples were provided by diploid healthy men, and the analysis parameters met the current World Health Organization standards. The DNA content was assessed by analysis of the corresponding spectra obtained from a laser confocal Raman spectroscope. The sperm sex chromosome information was obtained by fluorescence *in situ* hybridization (FISH). Comparative analysis was performed between FISH results and Raman spectral analysis results.

**Results:**

Different parts of the sperm head showed different spectral signal intensities, which indicated that there were different chemical components. Standard principal component analysis (PCA) can preliminarily classify sperm with different DNA contents into two groups. Further analysis showed that there were significant differences in the 785 DNA backbone peaks and 714–1,162 cm^−1^ DNA skeleton regions among sperm with different DNA contents. The peak and regional peak of the DNA skeleton of X sperm were significantly higher than those of Y sperm (X vs. Y, *p* < 0.05). The above sperm types were confirmed by FISH. ROC curve analysis shows that there is a correlation between the Raman spectrum data and FISH results.

**Conclusion:**

Raman spectroscopy can identify X and Y sperms by analyzing the DNA content difference. However, the accuracy of the detection still needs to be improved. Nevertheless, Raman spectroscopy has a potential application value in the field of sperm aneuploidy detection and may even be used as a non-invasive predictor of sperm aneuploid state in preimplantation genetic testing (PGT-A).

## Introduction

Spermatogenesis is a highly complex biological process in which spermatogonia undergo two meioses to produce sperm cells and transform into mature sperm through a series of nuclear and organelle changes (De Jonge and Barratt, 2006). In the process of spermatogenesis, if once meiosis is wrong, the normal chromosome balance changes, which leads to sperm aneuploidy (Rieger, 1968; McFeely, 1993). With the advancement of molecular diagnostic detection technology, it has been found that the normal chromosomes of males are also important factors for obtaining normal embryos, and sperm aneuploidy is directly related to embryo quality, which can lead to the failure of embryo implantation (Jan et al., 2004; Speyer et al., 2010) or repeated abortion during embryo development (Rodrigo et al., 2010; Scott et al., 2014; Ramasamy et al., 2015). Above all, aneuploid sperm fertilization not only affects embryo qualities but also leads to oocytes being wasted.

Preimplantation genetic testing of aneuploids (PGT-A) has become an indispensable technology in the field of assisted reproductive technology (ART; Franasiak et al., 2014). Currently, PGT-A for aneuploidy can detect defects in embryo chromosomes and select euploid embryos for transplantation (Eccles et al., 2017; Wilch and Morton, 2018). PGT-A uses different methods to identify the aneuploidy of all chromosomes, including the fluorescence *in situ* hybridization (FISH), array comparative genomic hybridization (CGH), and single nucleotide polymorphism (SNP) haplotypes combined with next-generation sequencing (NGS; Chen et al., 2018; Hu et al., 2018; Zhou et al., 2018). Despite the high accuracy of PGT-A, the embryo safety is still under doubt because the embryos must be biopsied. To date, little is known about the safety of the test samples obtained, although the initial report claimed that the method had no negative effects (Jiang et al., 2020). Furthermore, PGT-A can only be used to test normal embryos but cannot increase the number of transplantable embryos. Therefore, the non-invasive aneuploidy detection of gametes, especially sperm, may be an important way to improve the efficiency of assisted reproduction (Ranjith et al., 2014). Although the existing FISH and NGS technologies can detect the aneuploidy of single sperm, the sperm which is detected can no longer be used in ART. FISH detection only estimates the frequency of chromosomal abnormalities, which cannot guarantee the normal chromosomes of sperm used in ART, and there is no non-invasive technology to detect sperm aneuploidy (Patassini et al., 2013). Therefore, there is an urgent need for a non-invasive, efficient, and sensitive technique to directly assess sperm aneuploidy.

Raman spectroscopy is a non-invasive technique that allows the biochemical analysis of cellular components. As a vibration spectroscopy technique, Raman spectroscopy can identify chemical moieties through specific spectral patterns and can observe molecular changes with high specificity (Fragouli et al., 2017; Munné, 2018). It provides a rapid, simple, repeatable, non-destructive qualitative, and quantitative analysis of multicomponent materials by using the inelastic scattering of light, which has received increasing attention in research and clinical laboratories (Eberhardt et al., 2015). As early as 2014, there was study on non-invasive sex assessment in bovine semen by Raman spectroscopy combined with PCA analysis (De Luca et al., 2014). It has been reported that Raman spectroscopy can identify aneuploidy and aneuploidy embryos through embryo culture medium (Liang et al., 2019). In addition, Raman spectroscopy has detected oxidative DNA damage and mitochondrial damage caused by ultraviolet radiation (Konrad Meister et al., 2010; Mallidis et al., 2011). Therefore, the Raman technique may have great potential in the non-invasive detection of aneuploidy in germ cells, especially sperm.

In this study, we detected the human sperm chromosome DNA content by Raman spectroscopy and compared the Raman spectra data of X and Y sperms confirmed by FISH. Data analysis indicated that there were significant differences between the X and Y sperms Raman spectra data. Our results suggest Raman spectroscopy has a broad clinical application prospect in non-invasive sperm detection. However, more accurate and non-invasive improvement for detecting sperm aneuploidy is still needed.

## Materials And Methods

### Semen Sample Collection

Semen samples were provided by three diploid healthy men after G-banded karyotype, and the analysis parameters met the current World Health Organization (WHO, 2010) standards. All the ejaculation sperm samples were obtained by masturbation after 2–7 days of sexual abstinence, followed by liquefaction (37°C, 30 min).

This study was approved by the Ethics Committee of Reproductive Medicine of Xiangya Reproductive Heredity Hospital of CITIC (LL-SC-2018-038). The written informed consent was acquired from all donors who voluntarily participated in the research. The present study was conducted in accordance with the principles of the Helsinki Declaration and medical ethics.

### Separation of Spermatozoa

The liquefied semen samples were placed in a centrifugal tube and centrifuged (Eppendorf 5810R, Germany) at 300 rcf for 15 min. A pipette was used to aspirate the supernatant and left the sediment at the centrifugal tube, and then, 1 ml phosphate-buffered saline (1 × PBS) was added to the centrifugal tube. After slowly whisking to mix and centrifuging at 300 rcf for 5 min, the semen samples were washed with PBS and recentrifuged at 300 rcf for 5 min. Aliquots of 1 μl of the sperm suspensions were coated on a glass slide (Fisher, Thermo Scientific), fixed in methanol/acetic acid (3:1) for 5 min, then cleaned by PBS, and air-dried.

### Optimization of the Spectral Acquisition Method

The stepwise optimization of the spectral acquisition system is displayed in the supplementary graph. The laser power was constant at 10 mw. Firstly, we found that scanning a point once in 0.5 s generated stronger signals than scanning three times in 0.2 s did, which provided a better signal-to-noise ratio and clearer spectrum (Supplementary Figure S1A). Furthermore, we found that 15 scans in a point obtained better spectra than scanning only once (Supplementary Figure S1B).

### Laser Confocal Raman Microscope

We carried out analysis using a laser confocal Raman system (DXR Laser Microscopic Raman, Thermo Science). The system was equipped with an Olympus BX41 microscope, 532 nm laser (10 mW), adjustable confocal pinhole, automatic platform for microscopic sampling, standard resolution grating, XYZ drawing stage, and CCD inspection tester. The aperture of the pinhole collected by spectroscopy was 25 microns. The Olympus X 100 objective lens was used. We used a 532 nm laser and 10.0 mW laser power, and its image pixel size was 0.6 μm. The Raman signals of the thole sperm heads were acquired in the standard mode with an exposure time of 0.5 s, and each single sperm sample was scanned 15 times according to the above results. The spectra were obtained in a spectral range from 60 to 3,400 cm^−1^, and the background control spectrum was reduced from each sample spectrum. The process of Raman analysis took approximately 15–30 min per sample. We collected the Raman spectra of more than 200 sperm heads.

### Fluorescence *in situ* Hybridization

We dropped 20 μl dithiothreitol (DTT) onto a glass slide and covered the coverslip, removed the coverslip quickly after 7 min, immersed the slides in 1X PBS for 2 min, and then dehydrated the slides in an ethanol series (75, 90, and 100%) for 2 min each. We put the glass slides an incubator at 37°C overnight. Because there was a significant difference in Y and X chromosome content, the sex chromosome of the sperm was determined and distinguished by FISH with the following commercial probes: the X chromosome (DXZ1, Xcen alphasatellite, SpectrumGreen) and the long arm of the Y chromosome (DYZ1, Yq12 satellite III, SpectrumRed), which were purchased from Abbott-Vysis (Downers Grove, IL, United States). After hybridization and washing, the glass slides were stained using distamycin A/4(,6-diamidino-2-phenylindole; DAPI, Millipore, Temecula, CA, United States), observed under the Olympus BX51 fluorescence microscope (Olympus, Tokyo, Japan), and utilized to capture fluorescence signals and photograph the images with VideoTesT FISH 2.0 (VideoTesT, St Petersburg, Russia; Carmen et al., 2019). The sperm cells analyzed by Raman spectroscopy matched with FISH analysis one by one. Firstly, we marked the reverse side of the slide in the scanning area of Raman microscope with a diamond pen and took photographs to record the shape of sperm distribution under the Raman microscope. After Raman scanning and FISH procedure, we found the labeled area and obtained the FISH results under the fluorescence microscope. The Raman microscope figures were then compared with FISH figures to find the corresponding sperms. Due to the sperm loss during FISH procedure, only about one-third of the sperms analyzed by Raman spectrum were retained.

### Analysis of Raman Spectra

Before spectrum analysis, all the Raman spectral data were processed using the TQ analyst EZ version. First, the Savitzky–Golay smoothing data points 11 and polynomial (5 degrees) options were used to correct the baseline and subtract the spectral background (Supplementary Figure S2), and then, the spectrum was filtered by using a denoising algorithm (denoising option) to improve the resolution without losing spectral information. The signals of 714.30–1161.86 cm^−1^ (DNA-PO4 skeleton region) and 785 DNA skeleton peaks were used to present the sperm DNA content according to previous report (De Luca et al., 2014).

### Statistical Analysis

The measurements of all single sperm spectra data (over the 650–1,800 cm^−1^ spectral range) were grouped together and analyzed by standard principal component analysis (PCA) and were computed to acquire the corresponding PC vectors. Next, the scores concerning PC vectors were plotted singly in triangle and circle shapes for each group. Clustering according to the triangle and circle groups was observed in the score space diagram, suggesting that the sperm samples could be separated based on the spectra PCA analysis calculated using the MATLAB (R2010a) software system.

Data analysis was performed using SPSS 23.0, and values of *p* ≤ 5% were considered to be statistically significant. The data used in PCA were used for frequency distribution statistics. According to the results of FISH analysis, the corresponding sperm Raman spectrum data were divided into group x (X sperm) and group y (Y sperm). Mann–Whitney U-test was performed to calculate significant differences between the two groups. Then, the X and Y groups were categorized as dummy variables, and receiver operating characteristic (ROC) curve analysis was performed to evaluate the prediction value using the area under the curve (AUC) and to calculate the sex chromosome cutoff value.

## Results

### Spectra and Mapping

**Figure 1:**
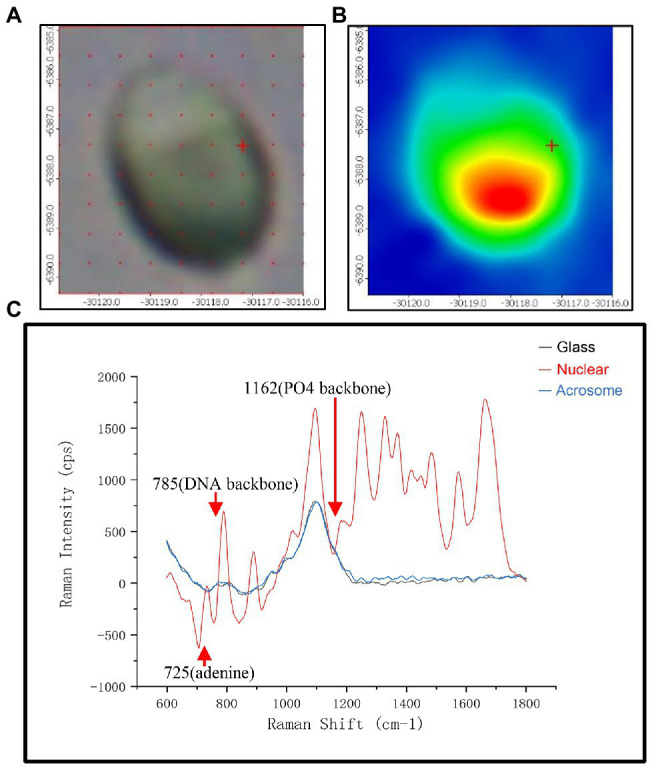
The spectra of different regions in the sperm head. **(A)** Microscopic confocal picture of a human sperm cell head fixed on the glass slide. **(B)** The phase diagram of Raman microspectroscopy of the sperm head corresponding to **(A)**. **(C)** Raman spectra of three different positions with glass (black), acrosome (red) and DNA at the sperm head. The x-axis units are displacement wavenumber (cm^−1^), nanometer (nm), and absolute wavenumber (cm^−1^). The y-axis unit is generally Raman strength.

**Figure 2:**
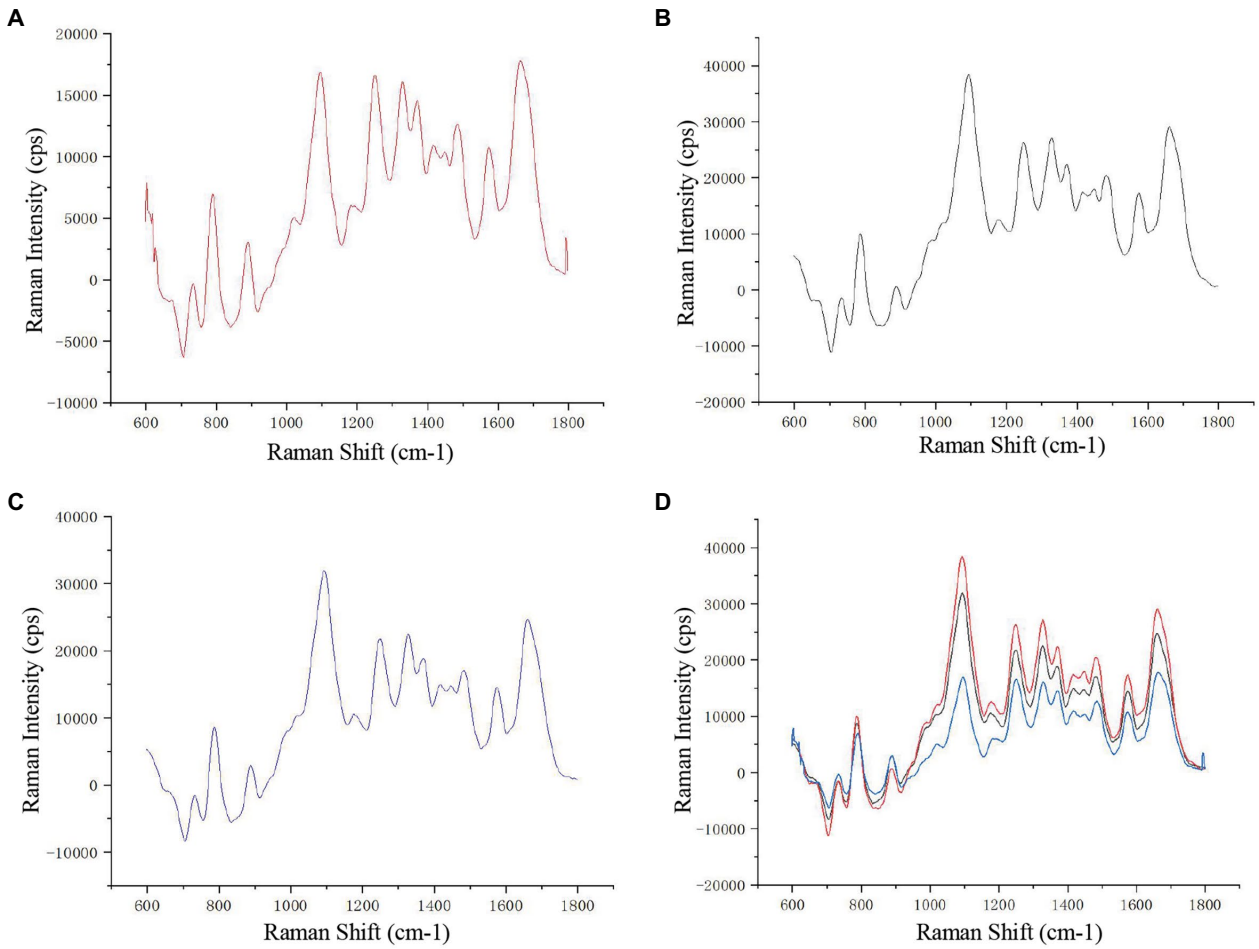
Raman spectra of different sperm heads. **(A–C)** Three single sperm head Raman spectra. **(D)** The combined Raman spectra of the three sperms.

To show the distribution of different chemical moieties clearly in the sperm head, we used the optimized final scheme to scan a single sperm. Four different colors of sperm head (blue, green, yellow, or red) were finally displayed, indicating the rich variety of chemical moieties in the sperm heads (Figures 1A,B). Then, we compared the Raman spectra of different parts of the human sperm head. The DNA skeleton, nucleotides, and phosphate skeleton were mainly located in the 725–1,162 cm^−1^ region. As shown in Figure 1C, the changes and intensities of Raman peaks of the glass (black), acrosome (blue), and nucleus (red) of sperm head were very different in this region. Next, to verify the specificity of the sperm nucleus Raman spectrum, we tested the other single sperm heads, which were very close to the previous sperm nucleus spectrum (Figures 2A–C). When the spectra of sperm were combined into the same profile, the spectral trends of the sperm were completely consistent, and only the peak values were slightly different, especially at peaks at 785, 1,095, and 1,250 cm^−1^ (Figure 2D).

### Raman Microspectroscopy and Sperm FISH

**Figure 3:**
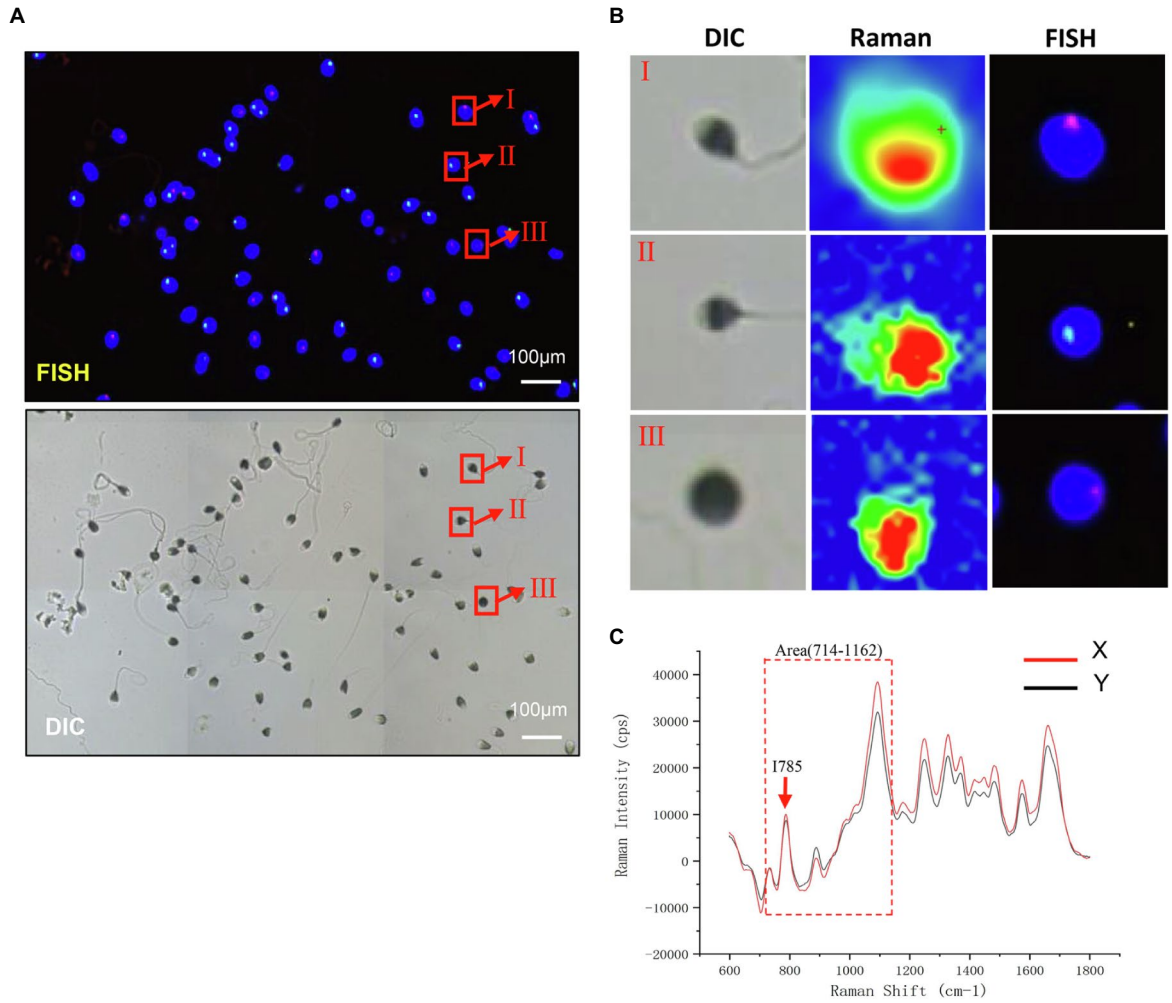
Raman light micrograph and corresponding FISH result map. **(A)** Analysis of sperm sex chromosome status in FISH under a fluorescence microscope. The nucleus of sperm head is stained by DAPI. The red hybridization signal on the blue sperm head represents X sperm, and green represents Y sperm (scale bar, 100 μm). **(B)** Planar Map of Raman Spectrum Corresponding to FISH (scale bar, 100 μm). **(C)** The Raman spectra of X and Y sperm. Red arrows, I785; Dashed box, Area (714–1,162).

To test the chromosomal difference of Raman scanning sperm, we subsequently performed FISH analysis with chromosome X and Y probes on the same slides. The distribution of X and Y sperm was approximately 2:1, as shown in Figures 3A,B. As shown in Figure 3C, the corresponding spectra of X sperm and Y sperm showed the same general characteristics and peak changing trend, but there were some differences in the corresponding DNA peaks. The peak intensity of X sperm at the DNA skeleton at 785 cm^−1^ and the PO4 skeleton at 1095 cm^−1^ was higher than that of Y sperm.

### PCA of the Spectra and Frequency Statistics

**Figure 4:**
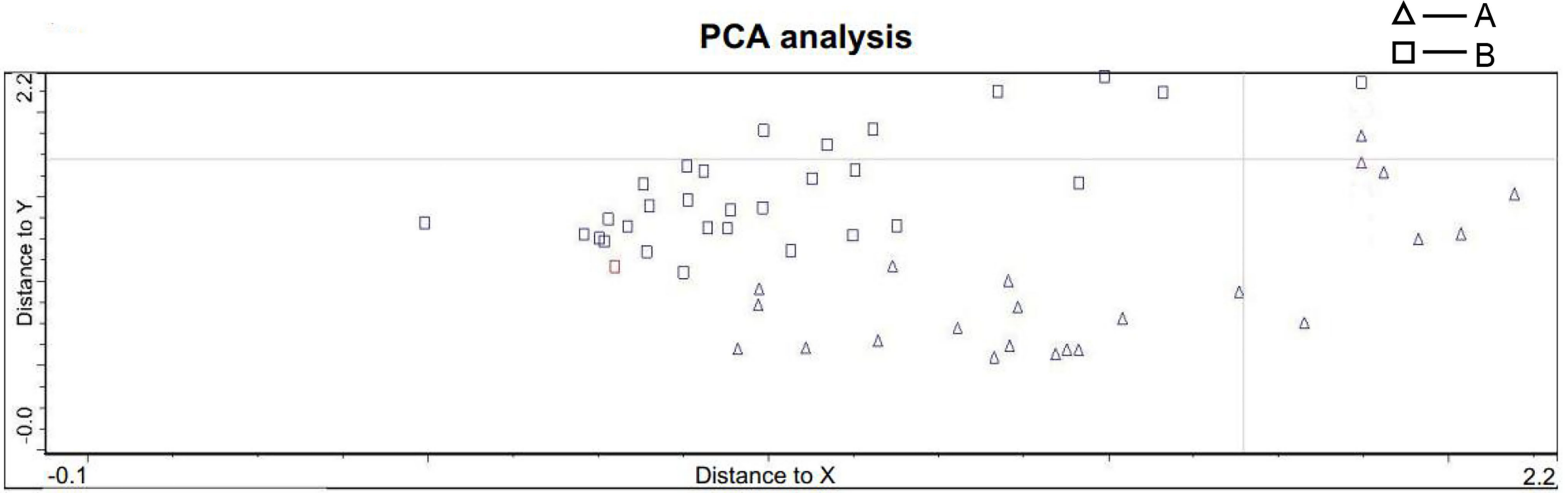
PCA statistics of sperm spectra. The sperm were divided into two groups: group A included 22 sperm, and group B included 31 sperm (A: triangle B: circle).

Due to the time-consuming nature of the Raman spectroscopy scan, we were able to scan 251 sperm with high quality and excellent signal. Most sperms were eluted during FISH processing, only 59 sperm had a one-to-one correspondence, including 39 X sperm and 20 Y sperm. The Raman spectra of each sample head were accumulated to obtain the average central spectrum. Among them, only 53 sperm were finally included in PCA (six sperms were excluded which could not be analyzed by PCA due to data deviation). These sperm were divided into two groups according to the calculation and calibration results of PCA: 22 sperm in group A and 31 sperm in group B (Figure 4), which was not exactly corresponded to the result of FISH. This result indicated that PCA could not distinguish the sperm sex chromosome completely and accurately.

Then, we conducted frequency distribution histogram statistics on the 59 sperm data, and the results showed that there were obvious frequency difference trends in the frequency distribution at I785 = 23,750 and Area714–1,162 = 3,250,000. For 20 Y sperm, the value of I785 mainly existed in the range of 17,000–23,000, and the mean value and median value were 21749.8 and 22,321, respectively. The main value range of Area (714–1,162) was 3,400,000–3,500,000, and the mean value and median values were 3,091,373 and 2,981,509, respectively. For 39 X sperm, the value of I785 mainly existed in the range of 29,000–35,000, and the mean value and median value were 26260.92 and 27,242, respectively. The main value range of Area (714–1,162) was 3,400,000–3,500,000, and the mean value and median value were 3,828,693 and 4,143,128, respectively. The analysis results indicated that these values could be the critical values of the difference between X and Y sperm (Supplementary Figures S2A–D).

### Discrimination of the Sperm With Different DNA Contents by Raman Spectroscopy

**Figure 5:**
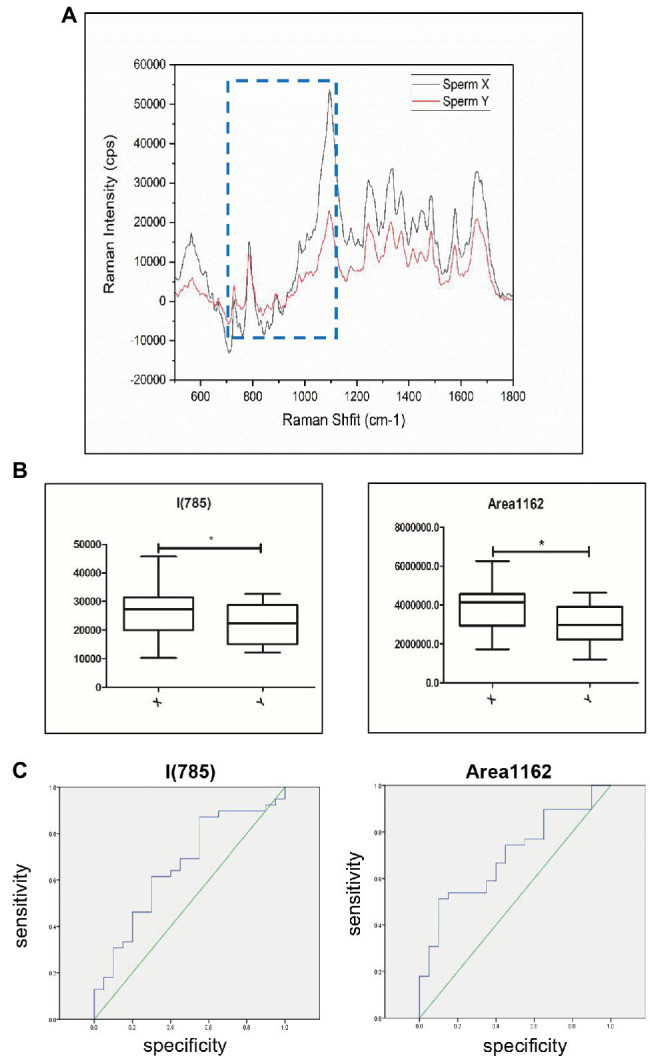
Discrimination of sperm with different DNA contents by Raman spectroscopy. **(A)** Raman spectra of the 500–1800 cm^−1^ area corresponding to X sperm and Y sperm. Black shows Y sperm and red shows X sperm. Raman peaks with the most significant difference for this study are highlighted by blue dotted lines. The x-axis units are displacement wavenumber (cm^−1^), nanometer (nm). and absolute wavenumber (cm^−1^). The y-axis unit is generally Raman strength. **(B)** The peak values of X and Y sperm in Area714-1,162 and I785. X, X sperm; Y, Y sperm. [*n* (X) = 39, *n* (Y) = 20, *, *p* < 0.05]. **(C)** Raman analysis data characteristics curve for sperm sex chromosomes. The area under the ROC curve indicates the prediction capacity of the model. Raman analysis data in I785 (AUC¼0.662). Raman analysis data in Area714-1,162 (AUC¼0.696).

The total Raman spectra showed that there were significant differences between X sperm (*n* = 39) and Y sperm (*n* = 20) at 714–1162 cm^−1^ and I785 (Figure 5A). Because the distribution of X and Y sperm data frequency does not accord with the normal distribution (Supplementary Figures S3A–D), we further analyzed the sperm of 59 cases by Mann–Whitney U-test rather than Student’s t-test. The values of X and Y sperms in I785 (*p* = 0.044) and Area (714–1,162; *p* = 0.0136) were statistically different. Compared with the Y sperm group, the peak values at 785 and 714–1,162 cm^−1^ in the X sperm group were higher than those in the Y sperm group, and the difference was more obvious in the regional peak (Figure 5B). ROC curve analysis was used to evaluate the sensitivity of the correlation between sperm DNA content and Raman spectra. The results showed that the corresponding thresholds of I785 = 24986.5 and Area714–1,162 cm^−1^ = 3,748,990 were the best for distinguishing the two kinds of sperm. When the peak value of 785 or 714–1,162 cm^−1^ exceeds this value, the possibility of X sperm is greatly increased. The AUCs of the ROC curves in both cases were 0.662 and 0.696, respectively (Figures 5C). Our results indicated that sperm DNA content has potential applicable value in the detection of sperm aneuploidy.

## Discussion

Human sperm cells are usually divided into the head and the tail, including connecting pieces (neck) and flagella. The head is usually oval, with a length of 4–5.5 μm, a width of 2.5–3.5 mm, and an aspect ratio of approximately 1:1.5. The top of the head is its acrosome area, covering approximately 40–70% of the head. The nucleus is located in the back of sperm head. It has been suggested that the aneuploidy of embryos may be caused by the fertilization of aneuploid sperm and oocytes, which may cause aneuploid blastocysts, resulting in implantation failure or loss of early pregnancy and waste of oocytes (Rodrigo et al., 2010; Scott et al., 2014; Arumugam et al., 2019). Although sperm FISH technology has become an important technology for male sperm aneuploidy detection (Ranjith et al., 2014), some shortcomings of the technology hinder its clinical application (Ioannou et al., 2018). FISH only analyzes the percentage of aneuploidic spermatozoa, and sperm treated with FISH cannot be used for normal fertilization again. Currently, the only criteria of better sperms in IVF lab remain to the sperm intensity, motility rate, and morphology, which cannot represent the euploidy of the sperms. Therefore, non-invasive sperm aneuploidy detection is very important to reduce the oocyte waste rate and improve the success rate of ART (Jiang et al., 2020).

Raman spectroscopy is a promising method for non-invasive sperm aneuploidy detection. It uses the inherent characteristics of light, and its intensity can be adjusted without labeling. It can identify the biological components of biological samples without affecting the integrity of cell structure and function (Agarwal and Said, 2003; Practice Committee of American Society for Reproductive Medicine in Collaboration with Society for Reproductive Endocrinology and Infertility, 2008; Con et al., 2014). In addition, because it is coupled with confocal microscopy, it is possible to analyze single cells. These characteristics make this technique very suitable for harmlessly assessing the biochemical properties of chemical moieties (Practice Committee of American Society for Reproductive Medicine in Collaboration with Society for Reproductive Endocrinology and Infertility, 2008; Con et al., 2014). Laser confocal Raman spectroscopy has been widely used in various fields and has become a new hot spot in the ART field. Previous research has used this technology to detect embryo culture medium to evaluate the aneuploidy of embryos (Liang et al., 2019). In addition, it was used to analyze sperm morphology and sperm head composition and evaluate the state of nuclear DNA to identify DNA damage (Kubasek et al., 1986; Mallidis et al., 2011; Davidson et al., 2013). PCA was used to distinguish X- and Y-bovine sperm cells based on single-cell Raman spectra, which could have a highly significant impact on animal production management systems as well as genetic improvement programs in farm animals (De Luca et al., 2014). Above all, numerous studies have shown that Raman spectroscopy may offer an alternative to the existing methods, which avoids the clinical limitations of the existing methods.

In this study, we explored whether laser confocal Raman spectroscopy could be used as a potential non-invasive sperm chromosome aneuploidy detection technique in assisted reproduction. The total length of the X chromosome was 156 megabases (Mb), including 1973 genes, while that of the Y chromosome was 57 Mb, including 496 genes (Quintana-Murci and Fellous, 2001). Since X chromosome is much larger than Y chromosome, the DNA content of a normal haploid X sperm is slightly higher than that of a normal haploid Y sperm. In consideration of the difference in genetic material between the two kinds of sperm, we used laser confocal Raman spectroscopy to evaluate the DNA content of these two kinds of sperms. We used Raman spectroscopy to show the optical spectrum of the sperm head region. To compare the accuracy of different analysis strategy to evaluate the sperm head DNA, we calculated both the 714.30–1161.86 cm^−1^ (DNA-PO4 skeleton region) and the 785 DNA skeleton peaks. Statistical analysis showed that there were significant differences between X sperm and Y sperm in the 714.30–1161.86 cm^−1^ (DNA-PO4 skeleton region) and 785 DNA skeleton peaks. Moreover, the 714.30–1161.86 cm^−1^ region spectral intensity was more accurate than 785 DNA skeleton peak intensity in distinguishing X and Y sperms. Our results showed that the Raman spectroscopy could distinguish the two kinds of sperms with different DNA contents. The difference of the chromosomal DNA content between aneuploid sperms and normal sperms is larger than that between X and Y sperms. According to this study, Raman spectroscopy combined with data analysis can screen out aneuploid sperms to avoid aneuploid embryos in ART.

However, due to the limitation of the current Raman spectral microscope, we can only collect the spectra of multiple fixed points of the sperm head instead of the whole sperm head spectra. This operation may miss the Raman spectra information of other sperm head regions, which may affect the accuracy of Raman analysis results. These may be the reason that the predicted X-Y sperm DNA difference is about 3%, while the X sperm spectral intensity of I785 and area (714–1,162) was 17 and 19% higher than that of Y sperm, respectively.

In our experiment, FISH process needed DNA denaturation, which resulted in dramatic sperm loss and reduced the number of corresponding sperms that could be analyzed. On the other hand, FISH with only X and Y numeric probes cannot detect the aneuploidy of other chromosomes, which may also affect the accuracy of the final results. Low-pass whole genomic sequencing may be a better approach to confirm the sperm aneuploidy status.

It remains challenging for quantitative analysis using Raman spectroscopy accurately to detect the unbalanced translocation of chromosomes, there is no consummate data analysis model to accurately analyze sperm Raman spectrum for accurate quantitative analysis, it is necessary to learn deeply to Establish a more perfect algorithm for data analysis. On the other hand, the current laser confocal Raman technology still uses the principle of infrared spectroscopy to detect the sperm, which must be washed and fixed on slides. These operations are still harmful to sperm and do not allow the sperm to be used for ART. Therefore, these methods cannot be used in clinical screening at present. In addition, the current Raman technology scanning is relatively time-consuming. The experimental steps and spectrum acquisition process still need to be further optimized to completely avoid sperm damage and improve efficiency. It is necessary to evaluate the safety of sperms under high-intensity laser before ART. In summary, the potential of Raman’s clinical application still needs to be further explored.

In conclusion, our research shows that Raman microscopic spectroscopy can identify sperm with different DNA contents. To our knowledge, this was the first report which analyzed the sperm DNA content and confirmed the results with FISH technology. Current Raman spectroscopy is time-consuming, hazardous to sperm due to long-term laser exposure, and fixation requirement. Development of sensitive Raman flow cytometry and microfluidic technology may overcome the above drawbacks and may have a potential application value in the field of sperm aneuploidy detection.

## Data Availability Statement

The raw data supporting the conclusions of this article will be made available by the authors, without undue reservation.

## Ethics Statement

The studies involving human participants were reviewed and approved by Institutional Review Board of the Reproductive and Genetic Hospital of CITIC-Xiangya. The patients/participants provided their written informed consent to participate in this study.

## Author Contributions

LH designed the research. ML and YJ performed research. DW and YZ performed the standard principal component analysis. HZ and GL analyzed the data. ML analyzed the data and wrote the paper. All authors contributed to the article and approved the submitted version.

## Funding

This research was supported by the National Key R&D Program of China (grant 2018YFC1003100, to LH), the National Natural Science Foundation of China (grant 81873478, to LH), the Hunan Provincial Grant for Innovative Province Construction (2019SK4012), and the Natural Science Foundation of Hunan Province (2018JJ6088, to YT).

## Conflict of Interest

DW and YZ were employed by company Thermo Fisher Scientific.

The remaining authors declare that the research was conducted in the absence of any commercial or financial relationships that could be construed as a potential conflict of interest.

## Publisher’s Note

All claims expressed in this article are solely those of the authors and do not necessarily represent those of their affiliated organizations, or those of the publisher, the editors and the reviewers. Any product that may be evaluated in this article, or claim that may be made by its manufacturer, is not guaranteed or endorsed by the publisher.

## Supplementary Material

Supplementary Figure S1 Optimization of spectral acquisition method. **(A)** Laser exposure time 0.5 s scanning once (red) and 0.2 s scanning three times spectrogram (black). **(B)** Laser exposure time 0.5 s scanning once and 15 times spectrogram. **(C)** Scanning map of a point in the sperm nucleus and its corresponding scanning position (scale bar, 100 μm). **(D)** The spectrogram of multiple sperm scans and corresponding Raman scans (scale bar, 100 μm).

Supplementary Figure S2 Original and processed spectra. Comparison between original spectrum and spectrum after automatic correction and smoothing by software (purple line: origin, green line: correction; red line: correction and smoothing).

Supplementary Figure S3 Frequency statistics of sperm spectra. **(A–D)** Frequency distribution histogram of 20 Y sperm and 39 X sperm data at I785 and Area (714–1,162); red dotted line: the potential critical value of the frequency distribution near the mean and median).
