# Supplementary figures and images for: Evaluation of Laser Confocal Raman Spectroscopy as a Non-Invasive Method for Detecting Sperm DNA Contents
Source: Front Physiol. 2022 Feb 8;13:827941. doi: 10.3389/fphys.2022.827941 (PMC8861532; doi:10.3389/fphys.2022.827941)

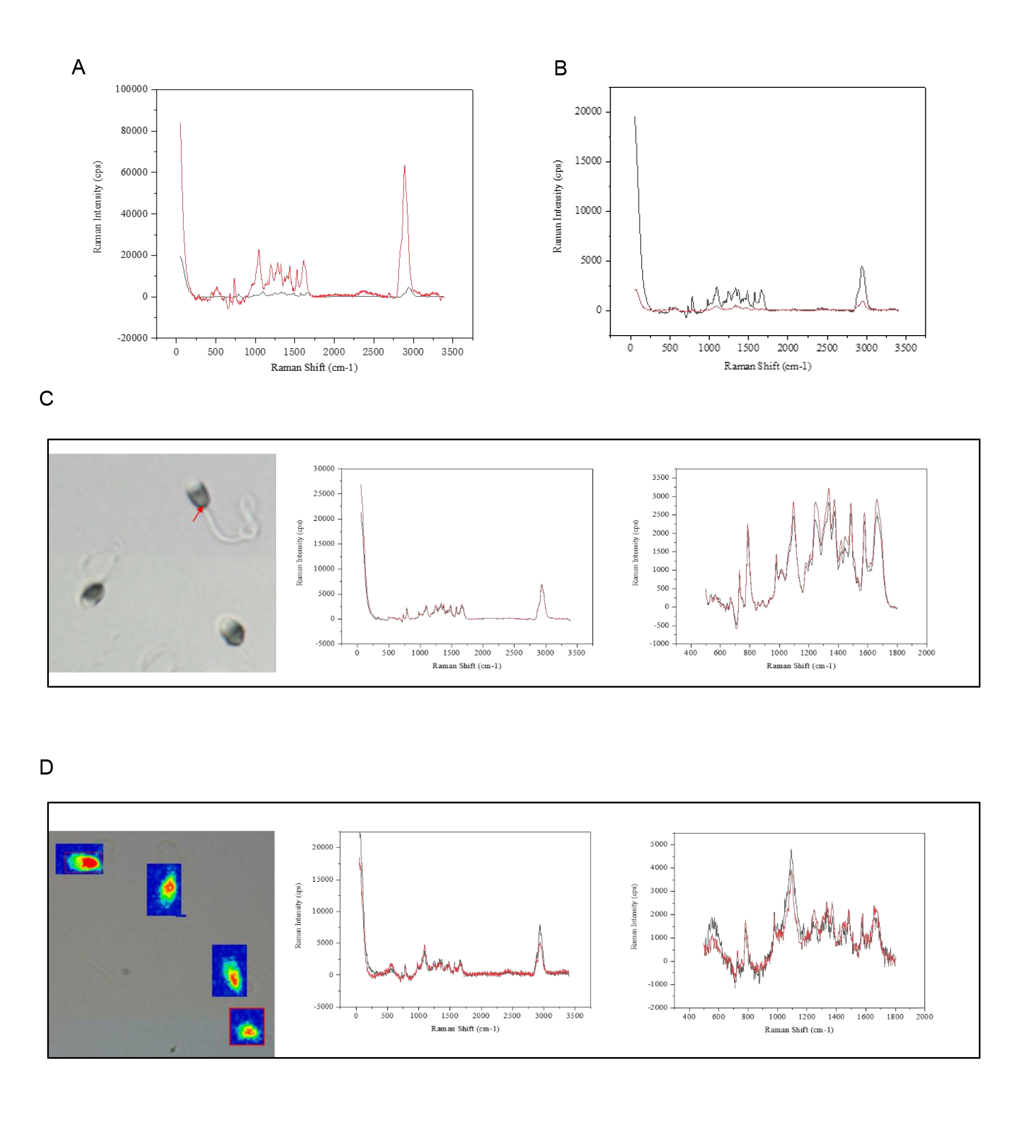

Supplement: Supplementary Figure S1 — Optimization of spectral acquisition method. (A) Laser exposure time 0.5 s scanning once (red) and 0.2 s scanning three times spectrogram (black). (B) Laser exposure time 0.5 s scanning once and 15 times spectrogram. (C) Scanning map of a point in the sperm nucleus and its corresponding scanning position (scale bar, 100 μm). (D) The spectrogram of multiple sperm scans and corresponding Raman scans (scale bar, 100 μm). [file Image_1.TIF]

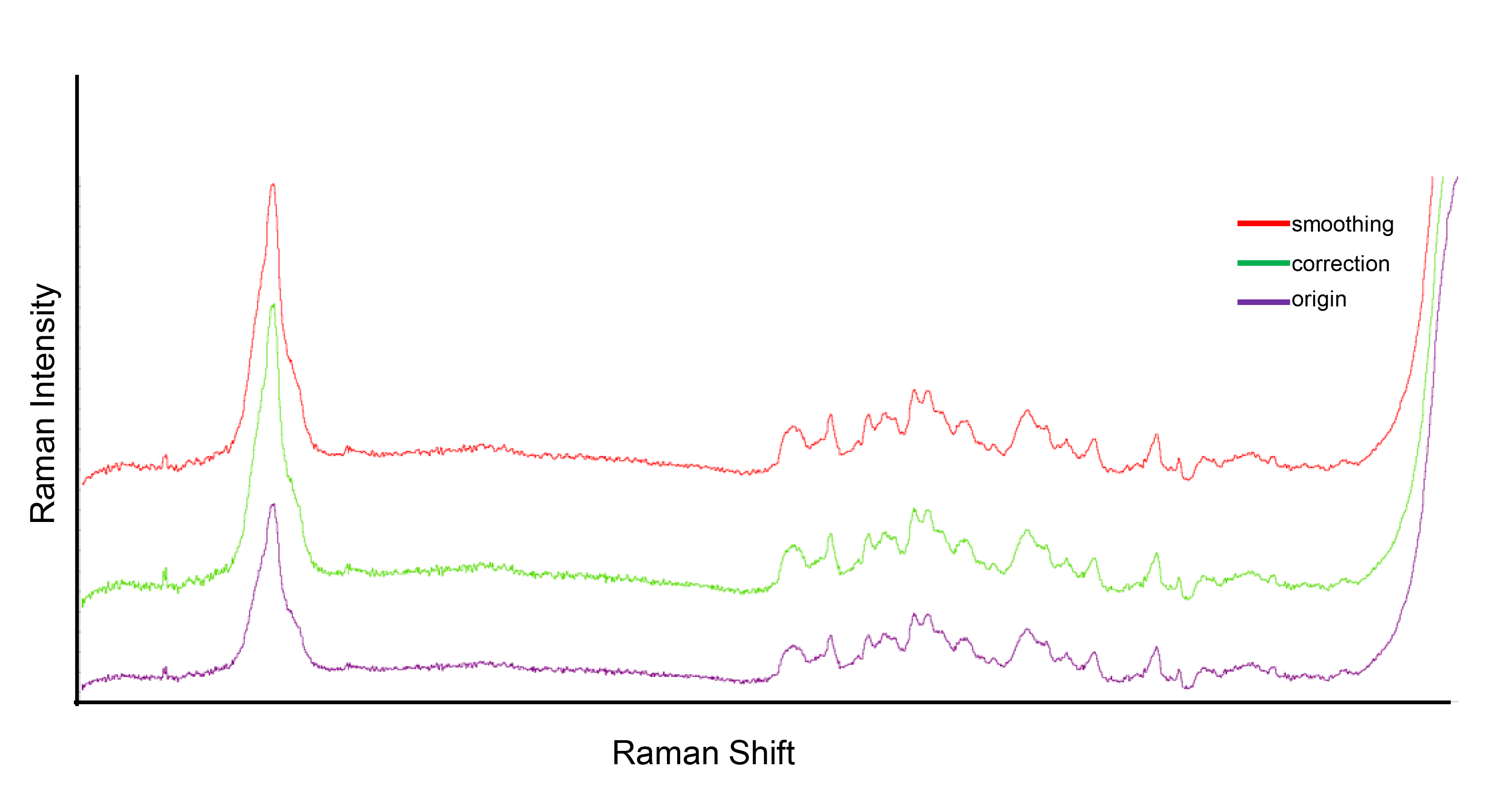

Supplement: Supplementary Figure S2 — Original and processed spectra. Comparison between original spectrum and spectrum after automatic correction and smoothing by software (purple line: origin, green line: correction; red line: correction and smoothing). [file Image_2.TIF]

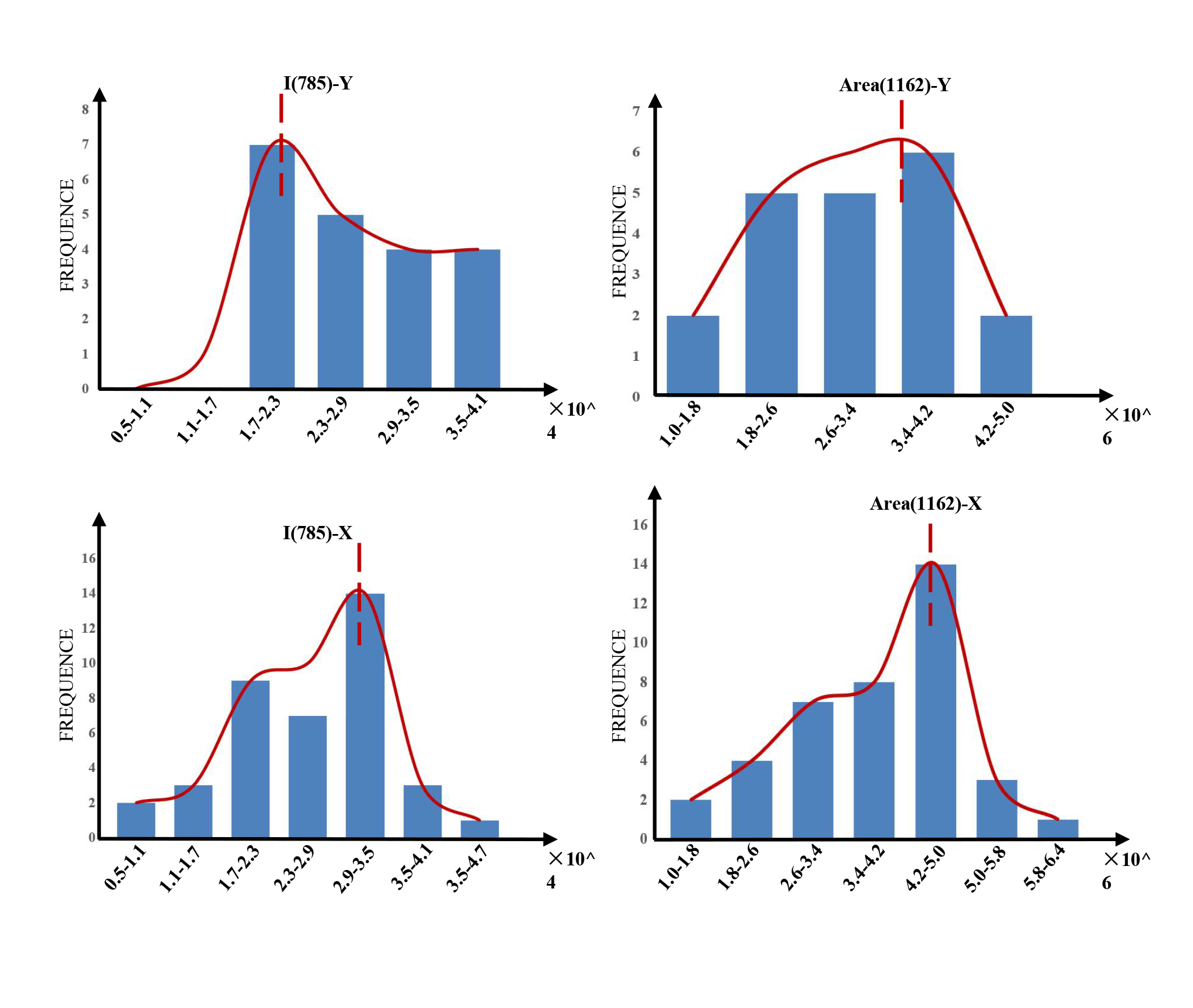

Supplement: Supplementary Figure S3 — Frequency statistics of sperm spectra. (A–D) Frequency distribution histogram of 20 Y sperm and 39 X sperm data at I785 and Area (714–1,162); red dotted line: the potential critical value of the frequency distribution near the mean and median). [file Image_3.TIF]
